# Resistance and Virulence Surveillance in *Escherichia coli* Isolated from Commercial Meat Samples: A One Health Approach

**DOI:** 10.3390/microorganisms11112712

**Published:** 2023-11-06

**Authors:** Maísa Fabiana Menck-Costa, Ana Angelita Sampaio Baptista, Matheus Silva Sanches, Beatriz Queiroz dos Santos, Claudinéia Emidio Cicero, Hellen Yukari Kitagawa, Larissa Justino, Leonardo Pinto Medeiros, Marielen de Souza, Sergio Paulo Dejato Rocha, Gerson Nakazato, Renata Katsuko Takayama Kobayashi

**Affiliations:** 1 Department of Microbiology, Center for Biological Science (CCB), State University of Londrina (UEL), Londrina 86057-970, Brazil; maisa.menckcosta@uel.br (M.F.M.-C.); matheus.silva.sanches@uel.br (M.S.S.); hellen.kitagawa@uel.br (H.Y.K.); leomedeiros27@gmail.com (L.P.M.); rochaspd@uel.br (S.P.D.R.); gnakazato@uel.br (G.N.); 2 Department of Preventive Veterinary Medicine, Center for Agricultural Sciences (CCA), State University of Londrina (UEL), Londrina 86057-970, Brazil; anaangelita@uel.br (A.A.S.B.); bqueirozds@gmail.com (B.Q.d.S.); claudineiaemidiocicero@gmail.com (C.E.C.); larissa.justino@uel.br (L.J.); marielen.souza@uel.br (M.d.S.)

**Keywords:** meat samples, ESBL, biofilm formation, virulence genes, extraintestinal *Escherichia coli*, diarrheagenic *Escherichia coli*

## Abstract

*Escherichia coli* is a key indicator of food hygiene, and its monitoring in meat samples points to the potential presence of antimicrobial-resistant strains capable of causing infections in humans, encompassing resistance profiles categorized as serious threats by the Centers for Disease Control and Prevention (CDC), such as Extended-Spectrum Beta-Lactamase (ESBL)—a problem with consequences for animal, human, and environmental health. The objective of the present work was to isolate and characterize ESBL-producing *E. coli* strains from poultry, pork, and beef meat samples, with a characterization of their virulence and antimicrobial resistance profiles. A total of 450 meat samples (150 chicken, 150 beef, and 150 pork) were obtained from supermarkets and subsequently cultured in medium supplemented with cefotaxime. The isolated colonies were characterized biochemically, followed by antibiogram testing using the disk diffusion technique. Further classification involved biofilm formation and the presence of antimicrobial resistance genes (*bla*_CTX-M_, AmpC-type, *mcr-1*, and *fosA3*), and virulence genes (*eae*A, *st*, *bfp*A, *lt*, *stx1*, *stx2*, *agg*R, *iss*, *omp*T, *hly*F, *iut*A, *iro*N, *fyu*A, *cva*C, and *hyl*A). Statistical analysis was performed via the likelihood-ratio test. In total, 168 strains were obtained, with 73% originating from chicken, 22% from pork, and 17% from beef samples. Notably, strains exhibited greater resistance to tetracycline (51%), ciprofloxacin (46%), and fosfomycin (38%), apart from β-lactams. The detection of antimicrobial resistance in food-isolated strains is noteworthy, underscoring the significance of antimicrobial resistance as a global concern. More than 90% of the strains were biofilm producers, and strains carrying many ExPEC genes were more likely to be biofilm formers (OR 2.42), which increases the problem since the microorganisms have a greater chance of environment persistence and genetic exchange. Regarding molecular characterization, bovine samples showed a higher prevalence of *bla*_CTX-M-1_ (OR 6.52), while chicken strains were more likely to carry the *fosA3* gene (OR 2.43, CI 1.17–5.05) and presented between 6 to 8 ExPEC genes (OR 2.5, CI 1.33–5.01) compared to other meat samples. Concerning diarrheagenic *E. coli* genes, two strains harbored *eae*. It is important to highlight these strains, as they exhibited both biofilm-forming capacities and multidrug resistance (MDR), potentially enabling colonization in diverse environments and causing infections. In conclusion, this study underscores the presence of β-lactamase-producing *E. coli* strains, mainly in poultry samples, compared to beef and pork samples. Furthermore, all meat sample strains exhibited many virulence-associated extraintestinal genes, with some strains harboring diarrheagenic *E. coli* (DEC) genes.

## 1. Introduction

Detection of resistance [1] and virulence [2] genes is pivotal in the characterization of microorganisms in food [3,4,5,6,7,8]. In addition, the ability of these microorganisms to survive in different environments, exhibiting heightened resistance to antimicrobial action [9,10], and their facile exchange of genetic material [11,12] contributes to effective monitoring and assists in the development of strategies to decrease the potential transmission of pathogens from food to humans.

Beef, pork, and poultry are among the main sources of protein in the human diet [13,14] and are correlated with most foodborne illnesses [15], thus serving as essential indicators of food safety [16,17]. Bacteria are responsible for 35.8% of foodborne illness cases in Brazil, according to the Brazilian Ministry of Health [18], and in the United States, they cause average annual costs of US $17.5 billion [19,20].

Food contamination may occur at any stage of the production chain, such as processing, washing, distribution, marketing, and even during home preparation [21,22,23]. Evisceration and skinning/plucking are critical points in the slaughter process [24,25,26]. Utensils and the handlers’ hands also contribute to spreading bacteria on the surface of carcasses and cross-contamination between them [23].

A method employed for assessing food contamination is through the analysis of hygiene indicator microorganisms [2,27], for example, *Escherichia coli*. This bacterium, a beneficial microorganism present in the gastrointestinal tracts of humans and animals, may harbor genes that confer pathogenic characteristics [28]. This microorganism is the major responsible for water and foodborne disease outbreaks in Brazil [29]. It can be classified into different pathotypes, depending on its isolation source, virulence factors, and associated clinical symptoms [28].

*E. coli* presents various diarrheagenic pathotypes (DEC), characterized by the presence of specific virulence genes. These include enteroinvasive *E. coli* (EIEC) a non-lactose fermenting pathotype, harboring the *ipa*H gene located on invasion plasmid H enteropathogenic; *E. coli* (EPEC) with the *eae* gene encoding intimin and maybe the *bfp* gene; enterotoxigenic *E. coli* (ETEC) identified by the *st* gene, which codes for a thermostable toxin, and the *lt* gene, responsible for a thermolabile toxin; Shiga toxin-producing *E. coli* (STEC) carrying the *stx* gene, which encodes the Shiga toxin; and enteroaggregative *E. coli* (EAEC) that presents aggregative adhesion in lineage cells, and often *agg*R gene, encoding aggregate adhesion fimbriae. These pathotypes can lead to a spectrum of clinical outcomes, ranging from mild diarrhea to conditions such as hemolytic uremic syndrome (HUS) and, in severe cases, death [28,30].

Extraintestinal pathogenic *E. coli* (ExPEC) has the potential to cause diverse infections, including meningitis, pneumonia, and urinary tract infections. Several genes facilitate the characterization of the virulence profiles in these strains, such as *iro*N (salmochelin siderophore receptor), *fyu*A (yersiniabactin siderophore receptor), *iut*A (aerobactin siderophore receptor), *omp*T (episomal outer membrane protein), *hly*A (hemolysin A), *hly*F (hemolysin F), *iss* (serum resistance-associated protein), and *cva*C (colicin V gene) [28,31]. Identifying the pathotypes of ExPEC present in meat samples is crucial [2], since these strains may be clonally related to those isolated from human infections [23,32].

Antimicrobial resistance in microorganisms isolated from meat samples poses a high risk to consumers [2,33]. Strains producing Extended-spectrum β-lactamase (ESBL), classified as serious threats by the Centers for Disease Control and Prevention (CDC), are responsible for over 35,000 annual deaths and two million infections [34]. Notably, during the COVID-19 pandemic, these numbers have surged by 32% [35]. ESBL-producing *E. coli* strains have been previously isolated from pork, poultry, beef, and other sources of animal protein [4,36,37,38,39,40,41]. Therefore, surveillance is pivotal in the context of the One Health framework.

Although the relationship between animal and human health dates back to the 19th century [42], the complexity of this connection led to the concept of ‘One World, One Health’ in 2003. This concept highlights the interconnection of humans, animals, plants, and their shared environment [43,44]. The existence of environments hosting diverse species raises pertinent health concerns [45], including issues related to food safety and antimicrobial resistance [46,47].

This study aimed to isolate and characterize ESBL-producing *Escherichia coli* strains obtained from chicken, pork, and beef meat samples, encompassing both phenotypic and genotypic evaluation of their virulence and antimicrobial resistance profiles.

## 2. Materials and Methods

### 2.1. Meat Samples

Over a consecutive five-month period (from August to December 2019), a total of 450 meat samples, comprising 150 each of chicken, beef, and pork, were acquired from supermarkets within the region of Londrina, Paraná State, Brazil. These samples, both commercial and refrigerated, were stored under refrigeration until processing.

### 2.2. Isolation and Biochemical Identification of E. coli

Meat samples were macerated and incubated in buffered peptone water (1:10—Difco, Detroit, MI, USA) at 37 °C for 18–24 h. After incubation, they were seeded onto MacConkey agar (Difco, Detroit, MI, USA) supplemented with cefotaxime (CTX) at a final concentration of 8 μg/mL. One lactose-fermenting colony per sample was subjected to biochemical identification using triple-sugar iron agar, indole production, Simmons citrate, urease production, lysine decarboxylation, and sorbitol and cellobiose fermentation tests [48].

These colonies were stored at −20 °C in Brain Heart Infusion (BHI) broth (Himedia Laboratories Pvt. Ltd., Mumbai, India), also supplemented with cefotaxime (8 μg/mL) and 20% glycerol until further processing.

### 2.3. Antibiotic Susceptibility Testing

Antimicrobial sensitivity was determined using the disk diffusion method [49], with seven distinct classes of antimicrobials: (1) β-lactams: ampicillin (AMP, 10 μg), amoxicillin-clavulanic acid (AMC, 10/20 μg), cefazolin (CFZ, 30 μg), cefoxitin, (CFO, 30 μg), ceftriaxone (CRO, 30 μg), ceftazidime (CAZ, 30 μg), cefotaxime (CTX, 30 μg), aztreonam (ATM, 30 μg), and imipenem (IPM, 30 μg); (2) quinolones: ciprofloxacin (CIP, 5 μg); (3) sulfonamides: sulfamethoxazole + trimethoprim (SXT, 1.25/23.75 μg); (4) tetracyclines: tetracycline (TET, 30 μg); (5) aminoglycosides: gentamicin (CN, 10 μg); (6) amphenicols: chloramphenicol (C, 30 μg); and (7) fosfomycin: fosfomycin/trometamol (FOT, 200 μg; Oxoid Ltd., Basingstoke, Hants, United Kingdom). The reference strain *E. coli* ATCC 25,922 was used as a control. Results were interpreted according to CLSI guidelines [50].

Multidrug resistance was characterized by resistance to three or more distinct classes of antimicrobial agents, excluding β-lactams [51].

### 2.4. Evaluation of Biofilm Formation

All isolates were submitted to quantitative biofilm formation assessment according to Stepanović et al. [52], using 96-well polystyrene plates and crystal violet staining. Absorbance readings (A) were measured at a wavelength of 540 nm using a spectrophotometer (LMR-961 plate reader—Loccus, ABIMAQ, São Paulo, Brazil) [53].

*E. coli* 042 (O44:H18) ATCC strain served as a positive biofilm formation control, while *E. coli* 25922 was the negative control. TSB broth (BD Difco, Franklin, NJ, USA) was used as a blank. Tests were performed in triplicate, and biofilms were categorized as absent, weak, moderate, strong, or very strong.

### 2.5. Molecular Identification of Resistance Genes

The following genes associated with β-lactam resistance were investigated: *bla*_CTX-M-1_, *bla*_CTX-M-2_, *bla*_CTX-M-8_, *bla*_CTX-M-9_, and *bla*_CTX-M-25_ [54], as well as AmpC-type production (MOX, FOX, EBC, ACC, DHA, and CIT) [55]. Moreover, the colistin resistance-coding gene (*mcr-1*) [56] and the fosfomycin resistance gene (*fosA3*) [57] were investigated.

### 2.6. Molecular Identification of Virulence Genes

The presence of genes related to DEC (*eae*A, *st*, *bfp*A, *lt*, *stx*1, *stx*2 [58], and *agg*R [59]), and genes associated with ExPEC (*iss*, *omp*T, *hly*F, *iut*A, *iro*N [60], *hyl*A, *fyu*A, and *cva*C [31]) was evaluated (Figure S4).

All polymerase chain reaction (PCR) amplicons were visualized using 1.5% agarose gels stained with GelRed (Biotium, Hayward, CA, USA).

After amplification of *eae* gene, by PCR, the products were purified with PureLink™ PCR Purification Kit (Invitrogen^®^ Life Technologies, Eugene, OR, USA) and sequenced using ABI3500 Genetic Analyzer sequencer, using BigDye^®^ Terminator v3.1 Cycle Sequencing Kit (Applied BioSystems^®^, Foster City, CA, USA).

The sequences were analyzed by Chromas (2.6.6) software and ClustalX (2.1), and using BLAST, the sequences were compared with the NCBI database.

### 2.7. Statistical Analysis

The odds ratio (OR) calculations were performed using the epiDisplay package (version 3.5.0.2). In this analysis, categorical data following a binomial distribution were modeled by logistic regression using the stats package (version 4.3.0). The variables were analyzed for their predictive values in OR calculation, and the statistical significance was determined via the likelihood-ratio test (LR-test) with a significance level of 5%.

For the analysis of ExPEC genes, isolates were grouped according to the number of genes they harbored: ExPEC 1 (0 to 2 genes), ExPEC 2 (3 to 5 genes), and ExPEC 3 (6 to 8 genes).

## 3. Results

### 3.1. Isolation and Identification of Strains Using Biochemical Methods

Out of the initial 450 samples (150 samples from each type of meat), subsequent to processing and identification, a total of 168 (37%) *E. coli* strains were isolated. Among these, 109/150 (73%—Figure S3) strains originated from chicken samples, 33/150 (22%—Figure S2) from pork samples, and 26/150 (17%—Figure S1) strains from beef samples.

Notably, chicken-derived strains were more likely to be ESBL producers, displaying the capacity of growth in culture media with third-generation cephalosporins, when compared to pork strains (OR 9.99, CI 5.74–17.80), and beef strains (OR 13.42, CI 7.50–24.73).

### 3.2. Antibiotic Susceptibility Testing

**Figure 1:**
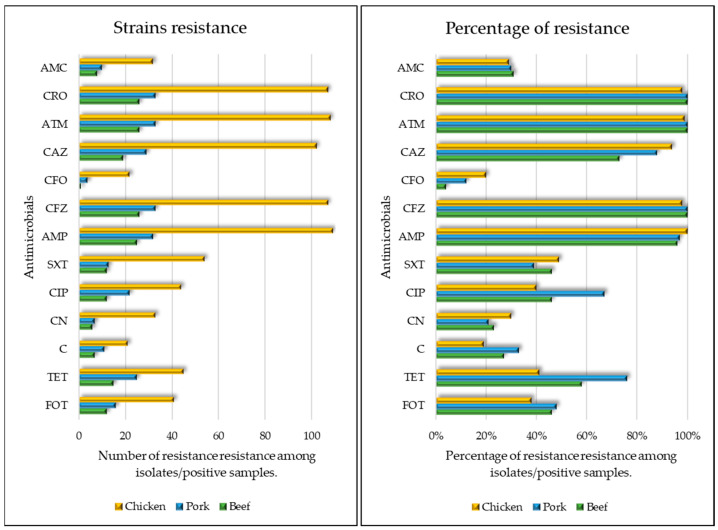
Comparative antimicrobial susceptibility profile across beef, chicken, and pork samples. On the left a figure relating to the number of isolates per meat sample and on the right a figure relating to the percentage of resistance per meat sample. FOT—fosfomycin-trometamol; TET—tetracycline; SXT—trimethoprim-sulfamethoxazole; C—chloramphenicol; CN—gentamicin; CIP—ciprofloxacin; AMC—amoxicillin-clavulanic acid; AMP—ampicillin; CFZ—cefazolin; CFO—cefoxitin; CRO—ceftriaxone; CAZ—ceftazidime; ATM—aztreonam.

Besides β-lactams, other noteworthy antimicrobial resistance patterns can be highlighted, with 51% (85/168) of strains exhibiting resistance to tetracycline, 46% (78/168) to ciprofloxacin, and 38% (64/168) to fosfomycin, while 100% of strains were susceptible to imipenem (Figure 1).

Furthermore, strains isolated from chicken samples presented a higher resistance rate to ceftazidime (OR 1.52, CI 1.21–1.91), while beef-derived strains displayed lower resistance to ceftazidime (OR 0.72, IC 0.60–0.87). Additionally, strains from pork were more resistant to tetracycline (OR 1.22, IC 1.07–1.40) and ciprofloxacin (OR 1.13, IC 1.01–1.28), when compared to the other antimicrobials.

Moreover, among all the strains, 45% (75/168) displayed resistance to three or more classes of antimicrobials, excluding β-lactams.

### 3.3. Evaluation of Biofilm Formation

It can be observed that only 6% (11/168) of all strains did not produce biofilms, while 46% (78/169) showed moderate biofilm production. Regarding different meat samples, 100% of the strains isolated from beef were biofilm producers (Table 1).

Additionally, strains harboring six to eight ExPEC genes (OR 2.42, CI 1.5–87) had a higher proportion of biofilm-producing strains compared to non-biofilm-producing strains.

### 3.4. Molecular Identification of Resistance Genes

**Figure 2:**
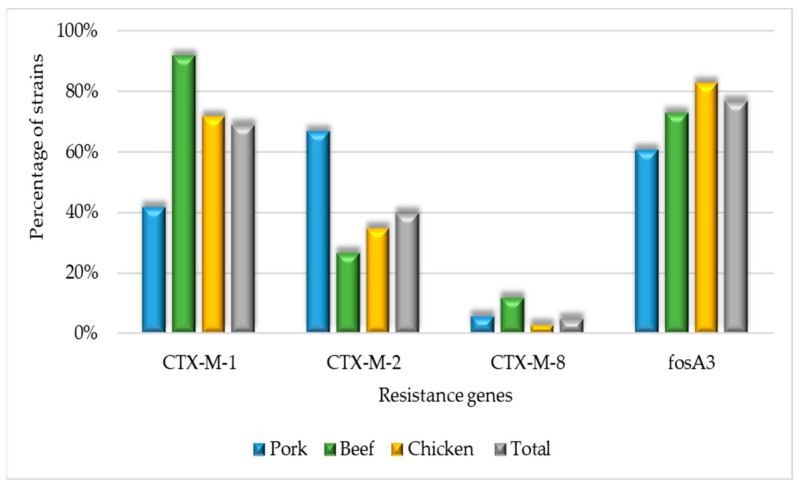
Molecular identification of resistance gene percentage across beef, chicken, and pork strains.

Regarding β-lactam resistance genes, they presented a lower prevalence of *bla*_CTX-M-9_ (OR 0.02, CI 0–0.37) than other genes within the *bla*_CTX-M_ group (Figure 2). No isolates harboring the *bla*_CTX-M-25_ and *mcr-1* genes were observed.

Strains from chicken were more likely to harbor the *fosA3* gene (OR 2.43, CI 1.17–5.05), while those from pork were less frequently associated with this gene (OR 0.37, CI 0.16–0.83) compared to other meat source. Beef-derived strains were more likely to carry the *bla*_CTX-M-1_ gene (OR 6.52, CI 1.48–28.72).

Among the Amp-C genes, it was observed that only the CIT gene was present in three strains, and no strains carried the MOX, FOX, EBC, ACC, or DHA genes. All the strains with the CIT gene were from chicken sources and displayed characteristics such as biofilm production, MDR, and the presence of *fosA3*, *iss*, *omp*T, *hly*F, *iro*N, and *iut*A genes.

### 3.5. Molecular Identification of ExPEC Virulence Genes

**Figure 3:**
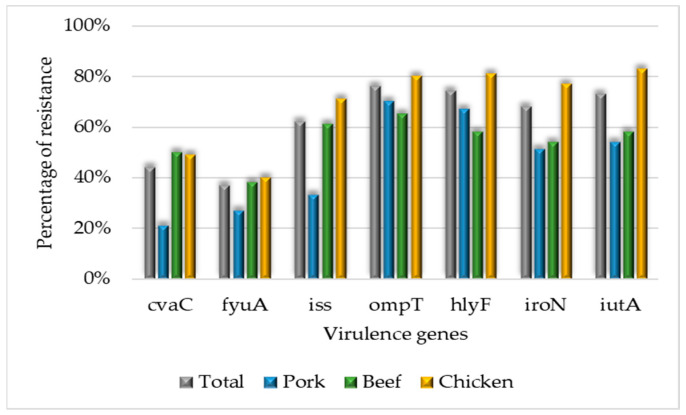
Percentage of virulence genes detected in beef, chicken, and pork strains.

The *hlyA* gene was not detected in any strain. The most prevalent gene was *ompT*, detected in 76% (127/168) of the strains, followed by *hly*F, *iut*A, *iro*N, *iss*, *cva*C, and *fyu*A (Figure 3).

Pork strains presented more ExPEC 1 (42%) and ExPEC 2 (33%). Chicken strains presented more ExPEC genes (six to eight ExPEC genes, ExPEC 3) (OR 2.5, CI 1.33–5.01) than other meat samples. Remarkably, strains carrying three to five ExPEC genes exhibited more resistance to CIP (OR 1.15, CI 1.01–1.32) than other antimicrobials (Table 2).

### 3.6. Features of Diarrheogenic E. coli (DEC) Strains

The results obtained for DEC genes demonstrated that all strains were negative for *stx*, *aggr*, *lt*, and *st*.

Additionally, two strains harbored the *eae* gene (confirmed by genetic sequencing) but lacked the *bfp* gene. One of these strains originated from pork, while the other was isolated from chicken. Both strains were MDR, harbored the *bla*_CTX-M-2_ and *fosA3* genes, and were classified as biofilm producers.

## 4. Discussion

In this study, we analyzed 450 samples, comprising 150 each from beef, pork, and chicken origins. The culture medium used in the initial isolation was supplemented with a third-generation cephalosporin (cefotaxime). Notably, poultry meat samples presented the highest number of *E. coli* strains, with more than 70% of samples yielding positive isolates, when compared to pork strains (OR 9.99, IC 5.74–17.80) and beef strains (OR 13.42, IC 7.50–24.73). Previous research conducted in the same region of the present study identified a high prevalence of MDR *E. coli* strains, ESBL producers, and carrying significant antimicrobial resistance genes in poultry production, as observed by Gazal et al. [61] and Menck–Costa et al. [48].

The detection of β-lactamase-producing strains is a major concern within the One Health approach [34,62,63]. According to the bulletin from the online notification system for hospital infections in Paraná [64], *Escherichia coli* was the fifth most frequently detected microorganism in diseases, with 31% of cases involving ESBL-producing strains. In our study, upon analysis of meat samples, we detected that 37% presented third-generation cephalosporins-resistant strains. But we alert that for chicken meat, 73% of carcass samples presented strains capable of growing in the presence of cefotaxime. It is worth highlighting that we are facing a pandemic of antimicrobial resistance [65].

Among the strains in this study, aside from being ESBL producers (exhibiting resistance to third-generation cephalosporins), over 45% were resistant to three or more classes of antimicrobials other than β-lactams. We detected greater resistance to tetracycline and ciprofloxacin, consistent with findings reported by Koga et al. [36] in strains isolated from chicken carcasses in Paraná in 2013, which showed high resistance rates to tetracycline and nalidixic acid. This suggests the persistence of a similar resistance profile over time.

The resistance profile to other antimicrobials among ESBL-producing strains was expected since the plasmids carrying CTX-M group genes often carry resistance determinants to other antimicrobials, as observed by Azargun et al. [66]. These researchers detected that approximately two-thirds of their ESBL-producing *Enterobacteriaceae* strains were resistant to quinolones. Similarly, Menck–Costa et al. [48] pointed out the potential of these strains to carry resistance to other antimicrobials, including quinolones, and correlated ESBL strains with the presence of the *fosA3* gene. These findings illustrate the importance of detecting and monitoring ESBL strains, especially in meat, as their presence can lead to transmission to humans through contaminated food, resulting in infections that may be difficult to treat [1,67].

In our study, the *fosA3* gene was most frequently detected in chicken meat samples (OR 2.43, CI 1.17–5.05) and least commonly in pork meat samples (OR 0.37, CI 0.16–0.83). Moreover, the high detection of resistance to fosfomycin in strains of animal origin is concerning, as this antimicrobial is considered the last therapeutic option against untreated urinary tract infections in humans [68,69]. Thus, selective antimicrobial use restricted to veterinary medicine or human medicine could minimize the problem of resistance in the animal-human axis [1].

It is essential to highlight that although this study identified a limited number of strains carrying DEC genes (specifically genes of atypical EPEC), these strains were classified as biofilm producers and displayed an MDR profile. Enteropathogenic *E. coli* (EPEC) can be characterized as typical or atypical. Typical EPEC strains possess the EAF plasmid, which includes the *eae* gene and is associated with the bundle-forming pilus (*bfp*) gene. This pathotype is known to cause diarrhea, particularly in children, while the atypical pathotype is frequently related to endemic outbreaks in underdeveloped countries [70]. Nevertheless, it is worth noting that the atypical EPEC, which lacks the *bfp* gene, is more commonly implicated in outbreaks in Brazil [29] and is rare in contaminated food sources [70,71].

The detection and prevention of DEC genes in such samples can help monitor the prevalence of DEC strains in the food supply. Furthermore, it is pivotal for the development of effective strategies to reduce their transmission to humans. Parussolo et al. [72] isolated strains of EPEC from raw milk in Brazil. These strains exhibited resistance to third-generation cephalosporins and carried the *bla*_TEM_ gene. The detection of DEC strains with ESBL genes in food samples increases the importance of surveillance efforts. DEC, which mainly affects developing countries [73], poses challenges for treatment owing to its antimicrobial resistance profile. It is interesting to point out that both strains harboring the *eae* gene did not present any ExPEC gene.

In this study, ExPEC 3—strains harboring six to eight genes of extraintestinal pathogenic *E. coli* were most detected in chicken meat (OR 2.5, CI 1.33–5.01). These strains are known to carry genes associated with Avian Pathogenic *Escherichia coli* [74,75]. The episomal outer membrane protease gene, *ompT*, which cleaves colicins, was the most prevalent among these strains. This gene has previously been detected in strains responsible for urinary tract infections in humans [76,77]. Soncini et al. [32] pointed out the existence of a clonal relationship between strains isolated from humans and those isolated from animal protein, highlighting the importance of monitoring strains originating from meat sources.

Furthermore, strains classified as ExPEC 3, possessing a high number of virulence genes, displayed greater biofilm-forming capacity (OR 2.42, CI 1–5.87), which worsened the problem since these microorganisms have an increased likelihood of persisting in various environments, facilitating genetic exchange [1]. Thus, they represent a substantial risk for causing extraintestinal infections in humans, as demonstrated by Cyoia et al. [4]. The possible exchange of antimicrobial resistance genes from environmental bacteria to bacteria associated with human infections remains a significant concern [1,23].

In the geographic region where this study was carried out raw consumption of beef, pork, and chicken is uncommon, but it is important to emphasize that cooking these food items effectively eliminates the microorganisms but does not eliminate their genetic material, with the potential capacity for incorporation by other viable microorganisms [78,79]. Additionally, these microorganisms can be spread horizontally within multiple environments, such as kitchens, butcher shops, and refrigerators, potentially contaminating other food. Importantly, some of these contaminated foods may not be cooked before consumption [80,81,82]. Consequently, the detection and characterization of *E. coli* strains isolated from meat samples are important for monitoring and crucial to maintaining public health and safety, reducing economic losses in the food industry, and ultimately enhancing food safety protocols.

Among ESBL strains, the *bla*_CTX-M-1_ gene was the most prevalent, consistent with findings from prior studies [48,83] and corroborated by a European study [84]. Remarkably, the predominance of CTX-M-1 genes was observed in beef samples, although beef is not typically regarded as a primary indicator of antimicrobial resistance [40,85,86,87]. Conversely, pork strains presented more *bla*_CTX-M-2_ genes, highlighting the critical importance of detecting and characterizing strains that produce β-lactamase enzymes, as they provide insights into the epidemiological distribution of these genes. Such insights hold significant relevance in the context of antimicrobial resistance, an issue that has consequences for animal, human, and environmental health.

The detection of AmpC-producing strains is pivotal as it evaluates resistance to β-lactams. The first AmpC, the CMY-1 gene, was reported by Bauernfeind in 1989 [88]. Over time, with increasing selection pressure, many AmpC β-lactamase genes, surpassing 220 types, have been reported. AmpC genes have been correlated with food-producing animals [89]. However, in our study, we detected only a limited number of AmpC-producing strains, all isolated from chicken. These strains exhibited multidrug resistance (MDR), biofilm-producing capacity, and harbored various resistance-associated genes, including *fosA3*, *iss*, *omp*T, *hly*F, *iro*N, and *iut*A. Therefore, strains with a relevant and worrying profile of antimicrobial resistance, as well as a high percentage of ExPEC genes, pose a risk for the occurrence of infections in humans, as discussed previously in this study.

All strains isolated in this study tested negative for carbapenem resistance, corroborating that carbapenem-resistant *Enterobacteriaceae* are not commonly found in non-human bacterial sources [38,83,90]. Moreover, the strains were also negative for the *bla*_CTX-M-25_ gene, which is commonly not detected in animal samples in Brazil [36,48,61]. Similarly, the *mcr-1* gene, responsible for colistin resistance, an important antimicrobial in human clinical use [91], was not detected in any strain.

Notably, the *hly*A gene, associated with alpha-hemolysin production and induction of kidney damage, was not detected in the strains. This gene is frequently isolated from patients with pyelonephritis [92] but has not been previously detected in meat strains during the same period [93].

According to Aslam et al. [94], antimicrobial resistance in samples of animal origin magnifies the emergence of superbugs. Several measures must be implemented to minimize this public health risk. These measures involve reducing the use of antimicrobials in animal production, ensuring meticulous care during animal slaughtering [95], imposing restrictions on the use of specific antimicrobials in animal production, highlighting the importance of these actions within the framework of the One Health approach, and ensuring the use of antimicrobials only under the guidance of professionals trained in the One Health approach.

The present study comprehensively characterizes *E. coli* strains resistant to third-generation cephalosporins (ESBL) in relation to relevant virulence and antimicrobial resistance genes. It underscores the public health risk these microorganisms pose in beef, chicken, and pork meat and highlights the importance of ongoing monitoring efforts.

## 5. Conclusions

Our study demonstrates the presence of β-lactamase-producing *E. coli* strains, mainly in poultry samples, when comparing different meat sources. Moreover, among all meat samples, ExPEC strains harboring many virulence genes, and some strains carrying DEC genes were identified. Notably, the samples exhibited a significant resistance profile to several classes of antimicrobials. These data corroborate existing literature and underscore the need for and importance of ongoing monitoring efforts for strains within meat samples.

## Figures and Tables

**Table 1:** Comparison of biofilm production profiles among isolates from beef, chicken, and pork samples.

Meat Samples/ Biofilm Producer	Pork 33	Beef 26	Chicken 109	Total 168
	*n* (%)			
Absent	4 (12.1)	0 (0)	7 (6.4)	11 (6.5)
Weak	16 (48.5)	19 (73.1)	25 (22.9)	60 (35.7)
Moderate	12 (33.6)	6 (23.1)	60 (55)	78 (46.4)
Strong	1 (3.0)	0 (0)	14 (12.8)	15 (8.9)
Very strong	0 (0)	1 (3.8)	3 (2.7)	4 (2.4)
Total	29 (87.9)	26 (100)	102 (93.6)	157 (93.4)

*n* = number of strains; % = percentage of biofilm production; Total = total number of biofilm-producing strains.

**Table 2:** Comparison of number of ExPEC genes profiles among isolates from beef, chicken, and pork samples.

Meat Sample/Number of ExPEC Genes	Beef 33	Pork 26	Chicken 109
	*n* (%)		
ExPEC 1	10 (38)	14 (42)	22 (20)
ExPEC 2	4 (15)	11 (33)	27 (25)
ExPEC 3	12 (46)	8 (24)	60 (55)
Total	26	33	109

*n* = number of strains; % = percentage of ExPEC genes; Total = number of strains per sample meat.

## Data Availability

The data presented in this study are available in Supplementary Materials.

## Supplementary Materials

The following supporting information can be downloaded at: https://www.mdpi.com/article/10.3390/microorganisms11112712/s1, Figure S1: Profile of *E. coli* strains isolated from beef meat samples; Figure S2: Profile of *E. coli* strains isolated from pork meat samples; Figure S3: Profile of *E. coli* strains isolated from chicken meat samples. Table S1: Primers used for the detection of diarrheagenic *E. coli* (DEC) and extraintestinal pathogenic *E. coli* (ExPEC) genes, as well as antimicrobial resistance-related genes.
