# Supplementary figures and images for: Resistance and Virulence Surveillance in Escherichia coli Isolated from Commercial Meat Samples: A One Health Approach
Source: Microorganisms. 2023 Nov 6;11(11):2712. doi: 10.3390/microorganisms11112712 (PMC10672981; doi:10.3390/microorganisms11112712)

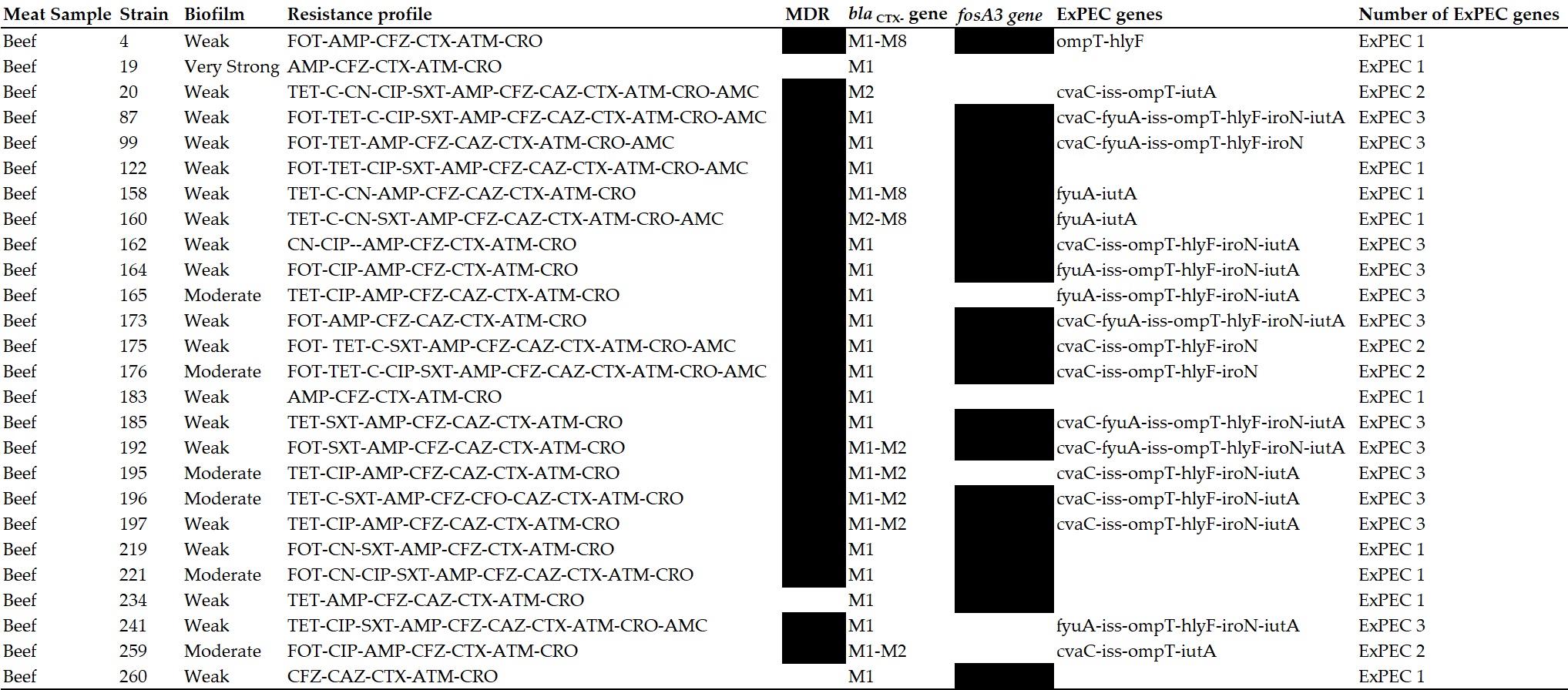

Supplement: Supplementary file 1 [file microorganisms-11-02712-s001.zip › Figure S1.jpg]

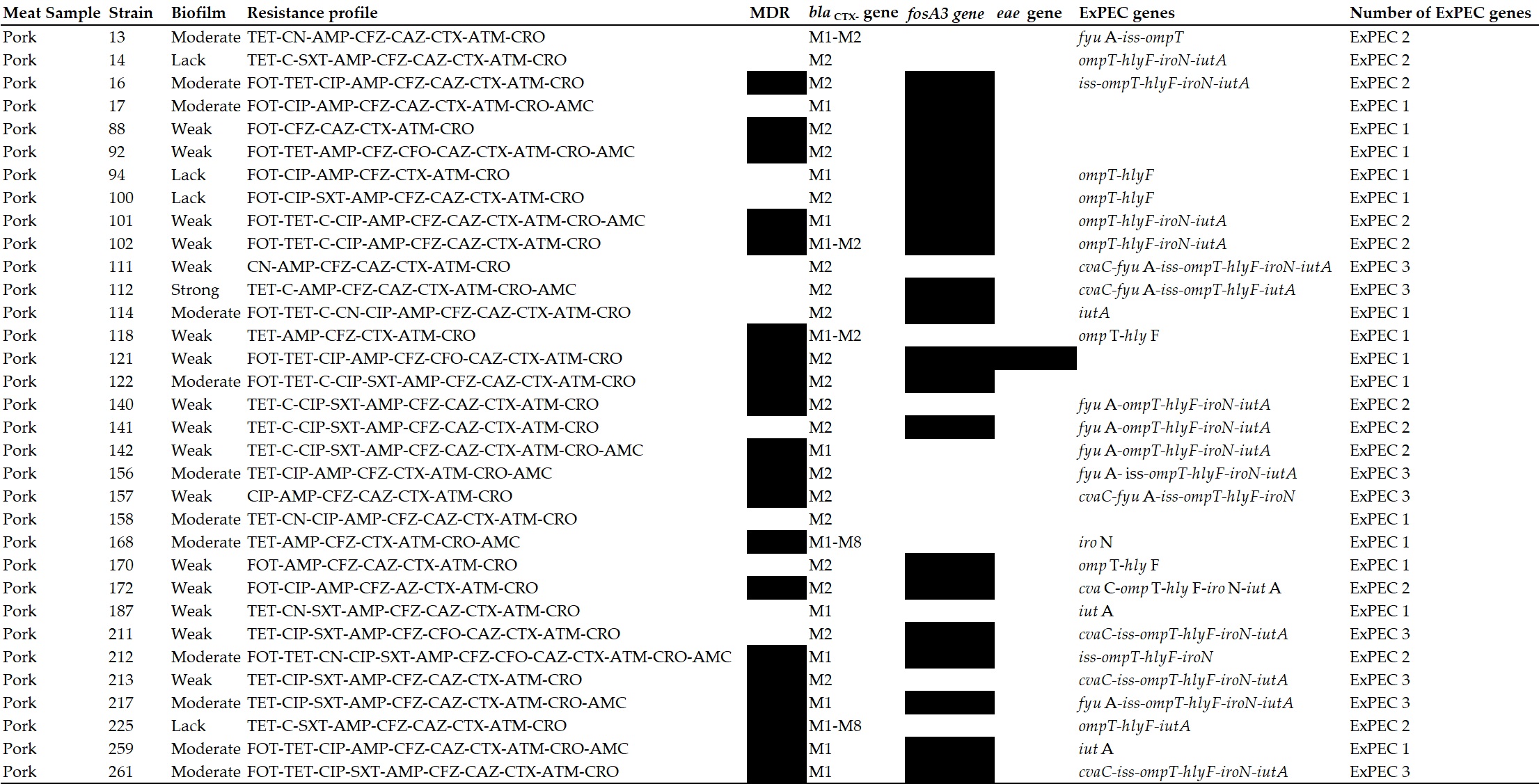

Supplement: Supplementary file 1 [file microorganisms-11-02712-s001.zip › Figure S2.jpg]

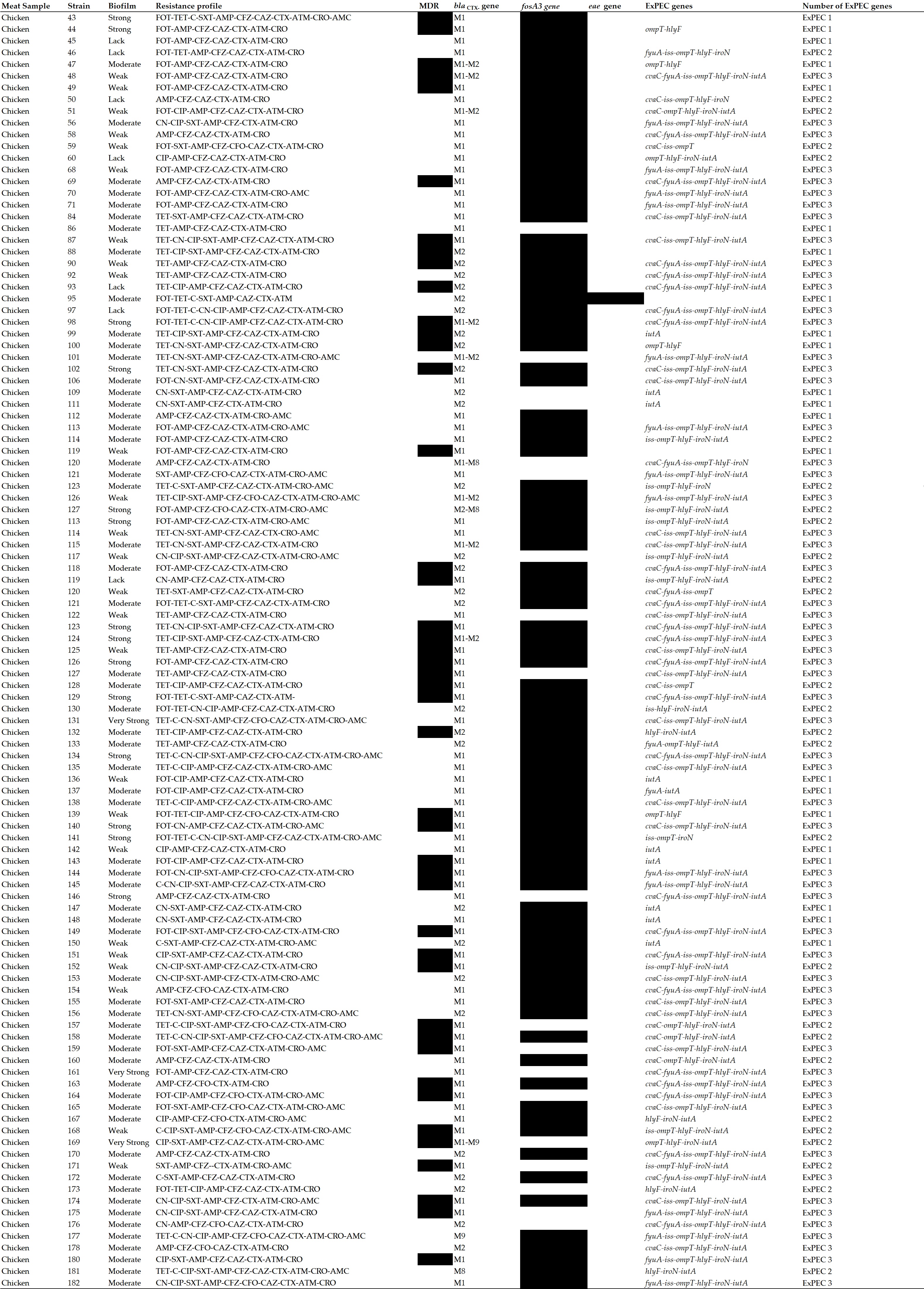

Supplement: Supplementary file 1 [file microorganisms-11-02712-s001.zip › Figure S3.jpg]

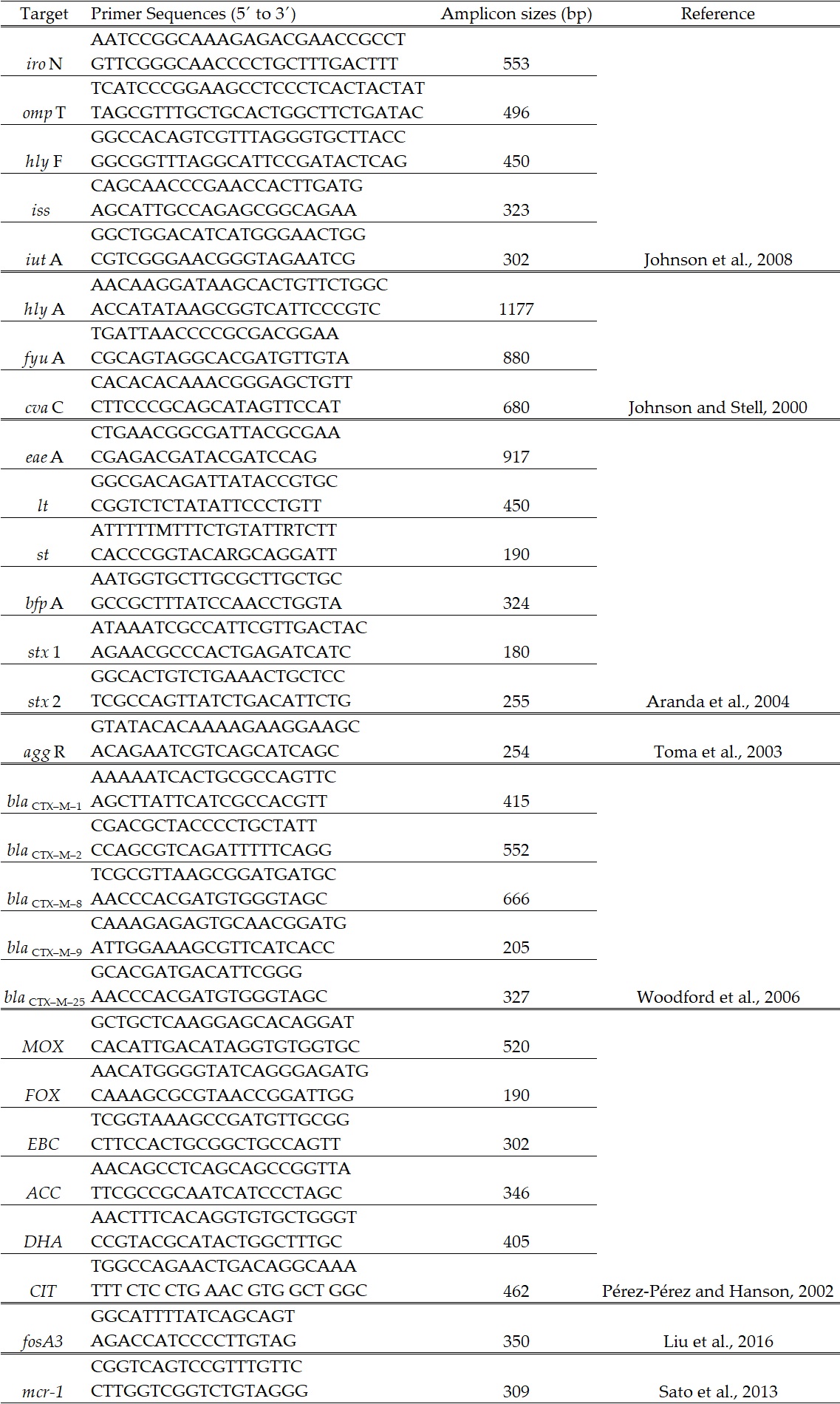

Supplement: Supplementary file 1 [file microorganisms-11-02712-s001.zip › Figure S4.jpg]
